# DeepAuC: Joint deep learning and auction for congestion-aware caching in Named Data Networking

**DOI:** 10.1371/journal.pone.0220813

**Published:** 2019-08-13

**Authors:** Anselme Ndikumana, Saeed Ullah, Do Hyeon Kim, Choong Seon Hong

**Affiliations:** Department of Computer Science and Engineering, Kyung Hee University, Yongin-si, Gyeonggi-do, Rep. of Korea; Chongqing University of Posts and Telecommunications, CHINA

## Abstract

Over the last few decades, the Internet has experienced tremendous growth in data traffic. This continuous growth due to the increase in the number of connected devices and platforms has dramatically boosted content consumption. However, retrieving content from the servers of Content Providers (CPs) can increase network traffic and incur high network delay and congestion. To address these challenges, we propose a joint deep learning and auction-based approach for congestion-aware caching in Named Data Networking (NDN), which aims to prevent congestion and minimize the content downloading delays. First, using recorded network traffic data on the Internet Service Provider (ISP) network, we propose a deep learning model to predict future traffic over transit links. Second, to prevent congestion and avoid high latency on transit links, which may experience congestion in the future; we propose a caching model that helps the ISP to cache content that has a high predicted future demand. Paid-content requires payment to be downloaded and cached. Therefore, we propose an auction mechanism to obtain paid-content at an optimal price. The simulation results show that our proposal prevents congestion and increases the profits of both ISPs and CPs.

## Introduction

In recent years, Internet traffic has continued to increase due to the growing number of connected devices with emerging platforms such as digital assistants, virtual, augmented, and mixed reality. It is estimated, by the year 2020, there will be 50 billion things connected to the Internet, which is equivalent to six devices per person [1]. This large-scale increase in both connected devices and platforms will tremendously increase content consumption. However, obtaining contents from Content Providers (CPs) can increase network traffic, which may cause a high network delay and congestion. Accordingly, caching content in close proximity to the users is one solution to reduce network traffic on transit links, i.e., bandwidth consumption [2–8].

Currently, many future Internet architectures, such as Content Distribution Network (CDN) [9] and Named Data Networking (NDN) [10], have been proposed to deal with network traffic increase by caching contents. The CDN is a distributed geographical network of caches or proxy servers that perform fast content delivery to users. In addition, CDN operates on existing TCP/IP architecture as an overlay architecture. Further, the CPs use cache-enabled servers of CDN providers to distribute their contents. Conversely, in NDN, users request contents by sending Interest packets and any node that has the requested contents can reply with Data packets. To route both Interest and Data packets, NDN uses content names rather than source and destination IP addresses [10]. In addition, inside the Internet Service Provider (ISP) network, routers in the transmission path can keep copies of the requested contents for satisfying future requests of the same contents. In other words, NDN permits the ISP to cache contents passing through its network [11], where user requests for contents can be served from cache storages of the routers. Therefore, caching may allow the ISP to reduce data traffic on transit links and prevent congestion, which can cause high network delays, packet losses, and retransmissions. Here, we chose NDN over other networks, such as CDN, because NDN allows the ISP to easily identify contents passing through its network using the content names.

As described in [12–14], we consider two categories of ISPs, the Access ISP and Transit ISPs. The Access ISP provides Internet access to end-users connected to its network on payment for access fees and using various technologies such as modems, and fiber optics. Conversely, Transit ISP also called Upstream Transit Provider has a larger network than Access ISP and connects Access ISP to the rest of the Internet in which Access ISP has no access itself. In other words, the Transit ISP has and sells large amounts of bandwidth that allows connecting Access ISPs to the rest of the Internet, where each Access ISP has to pay transit fee to the Transit ISP. Therefore, the Access ISP has a motivation to cache the contents for minimizing the transit traffic, i.e., the amount to pay for the transit traffic, and reducing the delays experienced by its customers in retrieving contents, where the Access ISP charges its customers the Internet access fees. Furthermore, as described in [12, 15], the Transit ISP does not have an incentive to cache the contents. However, the content owner or Content Provider (CP) can make an agreement with Access ISP or Transit ISP for content caching service. Moreover, the Transit ISP has large networks than Access ISP. Therefore, it is more reasonable that the CP makes a caching agreement with Transit ISP, as an incentive to participate in content distribution, rather than making it with Access ISP, which already has a motivation to cache the contents. Furthermore, the ISP has limited cache storage and deciding which contents to cache to prevent congestion is a challenging issue for the following reasons:

- First, the demand for content and the traffic volume on transit links vary considerably depending on the time and location. However, the Access ISP needs to cache contents that will have high future demand over congested transit links. This allows the Access ISP to prevent congestion by alleviating the traffic volume on the congested transit links and minimizes the network delays [11].
- Second, CPs make money through selling paid-contents, where users have to pay content prices/fees for using them [15, 16]. Therefore, allowing the Access ISP to cache paid content in its network, the CP will not have full control over its paid-content to track both content access and payment. In other words, the Access ISP will decide itself on paid-content placement inside its network to minimize transit bandwidth consumption and satisfy its customer demands without always contacting the CPs. To overcome these challenges, the authors in [17, 18] introduced the payment/remuneration from the Access ISP to the CP for the cached contents. However, when the Access ISP pays the content prices for downloading and caching them, this raises an issue on how the Access ISP can monetize its cache storages and obtain profits via caching paid-contents.
- Third, considering paid and non-paid-contents, paid-content requires a higher Quality of Service (QoS) than non-paid-contents to attract customers, and each customer needs to pay both the content price and Internet access fee [15]. Therefore, we need a caching approach that increases the profits of both the CPs and the Access ISPs.
- Lastly, because Transit ISPs gain more revenue when the transit traffic increases, there is no incentive for Transit ISPs to cache contents, unless they obtain an incentive from CPs for caching services. Conversely, Access ISPs have a greater incentive to cache content because content caching reduces the transit bandwidth payment and minimizes the delays experienced by their customers in retrieving contents [19]. Therefore, we need a caching approach that allows both Access and Transit ISPs to obtain benefits via caching contents.

To address the above challenges and deal with congestion, most of existing congestion control mechanisms detect congestion at an early stage and take reactive measures to prevent it. However, traditional congestion control algorithms cannot react immediately and this delay in reaction causes packet losses and increases network delays [20]. To address these issues, the authors in [20] and [21] introduced cooperative and distributed approaches for congestion control. The proposed approaches avoid network congestion before it occurs by monitoring the buffer size. Further, in NDN, both Pending Interest Table (PIT) and Content Store (CS) hits contribute in downsizing the number of Interest packets passing through the links [22]. However, in the above papers, the authors did not consider content caching as a way of preventing congestion in NDN. Accordingly, in Refs. [23] and [24], the authors proposed joint forwarding, caching, and congestion control approaches in NDN. The proposed approaches are used to maximize network utility and minimize network latency. However, demands for contents and the data traffic volume on transit links change depending on the time and location, and this issue was not considered in Refs. [23] and [24]. Due to the complexity of the problem of coordinating routing and caching, the authors in Ref. [25] showed that most existing protocols focused on the problem of forwarding demands for contents without considering the in-network caching scenario. To overcome this challenge, the authors introduced a new routing protocol that improves the cache hit probability. However, in [25], the authors have not considered the criteria to select contents to cache. Further, to prevent congestion before it occurs, methods reducing the sending rate or forwarding packets to another transit link(s) proposed in our previous work [20] can be used. However, in this paper, to prevent congestion before it occurs, we propose another technique that consists of caching contents that have higher predicted traffic volume and need to use transit links. We consider a prediction-based congestion control and content caching as proactive methods that can help to prevent congestion before it happens and avoid packet losses and delays experienced by existing reactive-based congestion control approaches. This new approach is more profitable for the Access ISPs than our previous approach and allows the Access ISP to minimize the transit bandwidth cost. In addition, for paid-content, using a joint caching and auction approach allows the Access ISP to obtain paid-content at an optimal price and participate in content selling by providing cached content to its customers on payment. The main contributions of this work are summarized as follows:

- First, we propose a deep learning model that helps the Access ISP to record transit traffic volume, learn the transit traffic characteristics, and predict the traffic volume that needs to be sent over the transit link(s). We chose deep learning model over other approaches discussed in the related works because network traffic is dynamic and this makes prediction more complex for mapping dynamic input with output. Therefore, to map dynamic input with output, deep learning model creates an abstract representation of network traffic as the traffic grows, automatically extracts features, and adjusts weights for predicting future traffic over transit links.
- Second, to prevent congestion and reduce the transit traffic volume passing through the congested transit links, we propose caching approach, where the Access ISP downloads and caches contents that have high predicted traffic volume over the transit link(s).
- Lastly, we consider that the Access ISP needs to pay the content price for downloading and caching paid-contents that have high predicted traffic volume. Therefore, to monetize cache storages and obtain profits from cached paid-contents, we propose an auction approach to all the Access ISP to obtain paid-content at an optimal price that minimizes its total payments. Based on the user demands, the ISP can sell the cached paid-contents. In other words, the Transit ISP can sell the cache contents to the Access ISP, while Access ISP can sell the cached contents to its users/customers.

Specifically, the novelties of our work over the related work are the following. (1) We consider the data traffic volume on the transit links to be varying over time and location. Based on the historical network traffic volume, the Access ISP can predict the contents that have high chance/probability of being requested over congested transit links. Then, the Access ISP caches such contents to reduce the transit traffic and minimize delays. (2) We consider that there are many types of contents that need to be treated differently during the caching process, that is, paid-contents require payment and higher QoS than non-paid-contents. (3) For paid-content, the Access ISP can make a profit by selling the cached contents, while for non-paid-content, the caching helps the Access ISP to minimize the transit bandwidth cost.

The remainder of this paper is structured as follows, Section 3 describes our system model, while Section 4 presents our joint deep learning and auction approaches for congestion-aware caching. Section 5 discusses the simulation results and analysis. Finally, we conclude the paper in Section 6.

## System model

**Fig 1:**
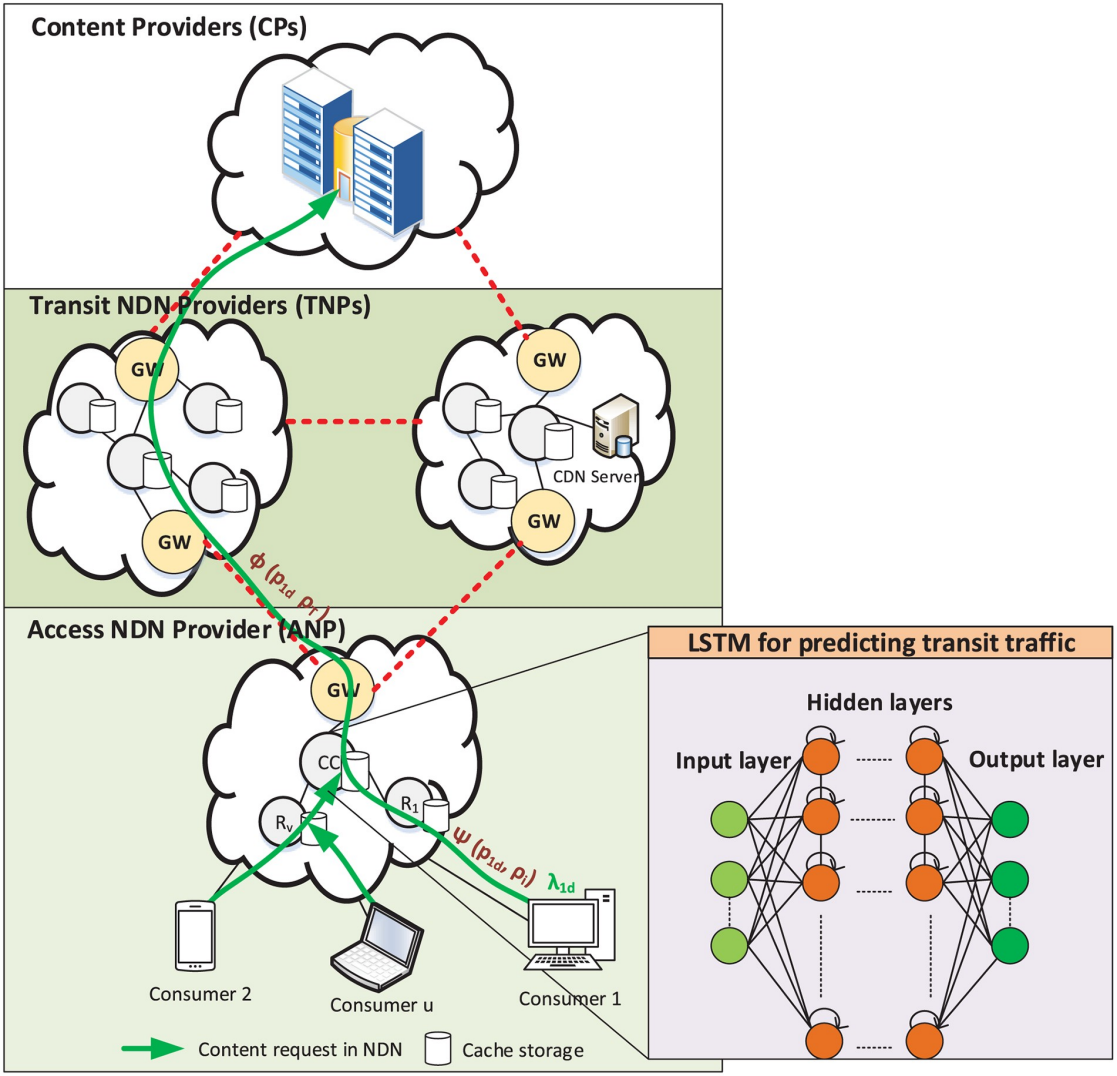
System model.

In our system model, demonstrated in Fig 1, we consider Access NDN Provider (ANP) as an Access ISP that provides Internet services to end-users/consumers. As summarized in Table 1 of key notations, we use an undirected graph G=(V,E) to model the ANP network. We consider V to be a set of *V* routers and E to be a set of *E* links. Among the routers of ANP, we have cached-enabled routers denoted R and Gateway Routers (GRs) denoted K, where V=R∪K. Each router r∈R is equipped with a cache storage of capacity *c*_*r*_. In addition, we denote I⊂E to be a set of *I* internal links (the black lines in Fig 1) in the ANP/ Transit NDN Provider (TNP) networks and T⊂E as a set of *T* transit links that connect the ANP network to the TNP networks (the red lines in Fig 1) such that E=I∪T. Further, we assume that the ANP is connected to one or more TNPs, where the TNPs provide Internet transit services to the ANP. Each internal link i∈I has a bandwidth of capacity *C*_*i*_ Mbps, while each transit link τ∈T has a bandwidth of capacity *C*_*τ*_ Mbps. Both the ANP and TNPs can serve the cached contents based on IP addresses, however, here, we consider the NDN traffic, i.e., the contents that can be retrieved by names rather than using the source and destination IP addresses. Here, for efficient name lookup, the approaches described in [26, 27] can be utilized, but we consider name lookup is outside the scope of this study.

**Table 1:** Summary of key notations.

Symbol	Definition
U	Set of consumers/users, |U|=U
R	Set of cache-enabled routers, |R|=R
*c*_*r*_	Cache capacity of each cache-enabled router r∈R
*p*_*c*_	Price per unit of cache storage
K	Set of gateway routers, |K|=K
V	Set of routers, |V|=V and V=R∪K
E	Set of links, |E|=E
I	Set of non-transit links, |I|=I
*C*_*i*_	Capacity of each internal link i∈I
T	Set of transit links, |T|=T
*C*_*τ*_	Capacity of each transit link τ∈T
N	Set of TNPs, |N|=N
D	Set of contents, |D|=D
W	Set of winners/TNPs for delivering paid-contents on payments
λ_*ud*_	Request from customer u∈U for content d∈D
*ρ*_*i*_	Volume of traffic in each non-transit link i∈I
*ρ*_*τ*_	Volume of transit traffic in each transit link τ∈T
$\rho_{\tau }^{\prime }$	Volume of predicted transit traffic in each transit link τ∈T
*γ*_*i*_	Internet access fee for internal link i∈I
*γ*_*τ*_	Transit fee per unit of bandwidth for each link τ∈T
*p*_*ud*_	Price that each consumer u∈U pays for accessing each paid-content d∈D
$p_{nd}^{*}$	Optimal price that the ANP pays to TNP n∈W
	for caching each paid-content d∈D
*θ*_*d*_	Delay tolerance threshold for each content d∈D
*β*_*d*_	Demand delay product for each content d∈D
*s*_*ud*_	Size of content d∈D requested by customer u∈U
*s*_*nd*_	Size of content *d* that can be supplied by each TNP *n*
*b*_*nd*_	Bidding value of TNP *n* for content d∈D

The GRs connect the ANP network to the TNP networks via transit links. Therefore, the content request/Interest packet for which the corresponding Data is not available/cached in the ANP network will arrive at the GR for transit. In such a situation, the GR sends out Interest packet to all transit links. The contents which are not cached inside the TNP can be retrieved from the servers of CP. However, the CP can collaborate with the TNPs to distribute the contents (using the cache-enabled routers of the TNP or CDN server). Here, agreements between the TNP and CPs for content distribution are outside the scope of this study. Hereafter, in this study, CPs are represented by TNPs.

We denote U to be a set of users and D to be a set of the contents. We assume that a request/ Interest λ_*ud*_ for content d∈D from consumer u∈U, which is a customer of the ANP, reaches GR k∈K if the content d∈D cannot be fetched from the ANP network (for example, the content requested by consumer 1). For such a request, using GRs, the ANP sends λ_*ud*_ to all transit links. Upon receiving λ_*ud*_, any TNP n∈N/ that possesses a copy of the requested content *d* in cache storage returns the content *d* on the reverse path of the Interest packet for free content.

The internal traffic volume that passes through each internal link i∈I is expressed as follows:

$$\begin{array}{c} \rho_{i}=\sum_{u\in U(i)}\sum_{d\in D}s_{ud}\lambda_{ud}h_{ud}\leq C_{i}, \end{array}$$ (1)

where U(i) is a set of the consumers that use an internal link *i* such that U(i)⊂U. In addition, *h*_*ud*_ ∈ {0, 1} is a cache hit indicator variable, where *h*_*ud*_ = 1 for the content d∈D returned to consumer u∈U from the cache storage of the ANP, and equals 0 otherwise. In addition, we use *s*_*ud*_ to denote the size of content *d* needed by user u∈U(i). Further, the total traffic of content *d* passing through the internal link *i* needs to respect the link capacity *C*_*i*_ constraint (1). However, the NDN architecture uses packet levels, which makes the content size unknown before it is received. To address this challenge, we use Frequency-Based Content Size (FBCS) described in our previous work [11] to find *s*_*ud*_.

For the transit link τ∈T, we use *ρ*_*τ*_ to denote the volume of the transit traffic, where *ρ*_*τ*_ is expressed as follows:

$$\begin{array}{c} \rho_{\tau }=\sum_{u\in U(\tau )}\sum_{d\in D}s_{ud}\lambda_{ud}\left(1-h_{ud}\right)\leq C_{\tau }, \end{array}$$ (2)

where U(τ)⊂U is the set of consumers that use the transit link *τ*. In addition, for the content *d* that is not available in the ANP network, 1 − *h*_*ud*_ is used as a cache miss indicator variable. Further, the total volume of the transit traffic for the content *d* that passes through the transit link *τ* needs to respect the transit link capacity constraint (2).

To prevent congestion on the transit links and to minimize delay, the ANP monitors its network traffic and keeps records of the historical traffic volume in the form of a traffic matrix (TM), as described in Ref. [28]. Then, the ANP uses the TM denoted *X*^*τ*^ to learn the transit traffic characteristics. Here, *X*^*τ*^ is a four dimensional matrix denoted *K* × *K* × *T* × *D*, where K is the number of GRs in the ANP and TNP networks, *T* is the time, and *D* indicates content names. In other words, *X*^*τ*^ is a tensor of *K* × *K* × *T* × *D* dimensions, where an entry $x_{kltd}^{\tau }$ indicates the number of packets for content *d* sent through the transit link τ∈T at each time slot *t*. Moreover, each outgoing face k∈K of the GR of the ANP is connected to the incoming face l∈K of the GR of TNP via the transit link τ∈T. Because, in NDN, contents are requested using names, we convert the content names into numeric values for ease of computation. Further, for TM prediction, we use Long Short-Term Memory (LSTM) recurrent neural networks [29], as described in Section. We chose LSTM over other prediction methods such as ARIMA and Prophet [30] because it has a cyclic connection over time and memory to temporally store the internal state information of the network. Moreover, our LSTM model is implemented in Content Controller (CC) proposed in Refs. [11, 31].

To prevent congestion by alleviating the traffic load on heavily used transit link(s) and to minimize the network delay, the ANP caches the contents that have high predicted demands. The ANP can obtain free contents without paying content fees. However, for paid-content, the ANP needs to pay *p*_*nd*_ to obtain and cache the paid-content *d*. For paid-content, based on user demands, the ISP sells the contents to customers, where each customer pays *p*_*ud*_ to obtain the content *d* (*P*_*ud*_ is the price of the paid-content *d*, while *P*_*ud*_ = 0 for free contents). Further, we assume that *p*_*ud*_ < *p*_*nd*_. More details concerning finding the optimal *p*_*nd*_ are provided in Section.

We use *f*_*d*(*τ*)_ as a cache decision variable that indicates whether or not the ANP caches the content d∈D that passes through the transit link τ∈T. *f*_*d*(*τ*)_ is given by:

$$\begin{array}{c} f_{d(\tau )}=\{\begin{array}{c} 1,\;\text{if}\;\rho_{\tau }\geq C_{\tau },\\ 0,\;\text{otherwise}. \end{array} \end{array}$$ (3)

The amount Φ(*p*_*nd*_, *γ*_*τ*_) that the ANP needs to pay to the TNP depends on the transit traffic volume $\sum_{\tau \in T}\gamma_{\tau }\rho_{\tau }$ and the contents that need to be cached. Φ(*p*_*nd*_, *γ*_*τ*_) is expressed as follows:

$$\begin{array}{c} \Phi \left(p_{nd},\gamma_{\tau }\right)=\{\begin{array}{c} \sum_{\tau \in T}\;\gamma_{\tau }\rho_{\tau }+\sum_{u\in U(\tau )}\sum_{d\in D}p_{nd}\lambda_{ud}\left(1-h_{ud}\right),\\ \text{if}\;\beta_{d}\geq \theta_{d}\;\text{and}\;f_{d(\tau )}=1\text{,}\\ \sum_{\tau \in T}\;\gamma_{\tau }\rho_{\tau }+\sum_{u\in U(i)}\sum_{d\in D}p_{ud}\lambda_{ud}h_{ud},\\ \;\text{otherwise,} \end{array} \end{array}$$ (4)

where *γ*_*τ*_ is the transit fee per unit of bandwidth. For each content *d*, we use *θ*_*d*_ > 0 to denote the delay tolerance threshold. Further, *β*_*d*_ denotes the demand delay product as defined in Ref. [11]. If *β*_*d*_ ≥ *θ*_*d*_ and *f*_*d*(*τ*)_ = 1, the ANP pays *p*_*nd*_ for caching the paid-content *d*. Otherwise, the ANP does not need to cache the paid-content *d* (*p*_*nd*_ = 0), i.e., the ANP only forwards the content price *p*_*ud*_ from its consumer to the TNP. The amount that the consumers pay to the ANP to obtain content and access bandwidth is expressed as follows:

$$\begin{array}{c} \Psi \left(p_{ud},\gamma_{i}\right)=\{\begin{array}{c} \sum_{i\in I}\;\gamma_{u}\rho_{i}+\sum_{u\in U(i)}\sum_{d\in D}p_{ud}\lambda_{ud}h_{ud},\\ \text{for}\;\text{paid-content,}\\ \sum_{\tau \in T}\;\gamma_{\tau }\rho_{\tau },\;\text{otherwise}. \end{array} \end{array}$$ (5)

The utility *U*_*A*_(*p*_*ud*_, *p*_*nd*_, *c*_*r*_) of the ANP is based on (*i*) the payment Ψ(*p*_*ud*_, *γ*_*i*_) from customers to the ANP for Internet access and for downloading the cached paid-contents; (*ii*) the payment Φ(*p*_*nd*_, *γ*_*τ*_) that the ANP pays to the TNP for both the transit bandwidth and the cached paid-content *d*; and (*iii*) the cost of cache storage deployment: $\sum_{r\in R}\;c_{r}p_{c}$. We use *p*_*c*_ as a price per unit of cache storage and *c*_*r*_ as the size of the cache storage of a cache-enabled router r∈R. *U*_*A*_(*p*_*ud*_, *p*_*nd*_, *c*_*r*_) is given by:

$$\begin{array}{c} U_{A}\left(p_{ud},p_{nd},c_{r}\right)=\Psi \left(p_{ud},\gamma_{i}\right)-\Phi \left(p_{nd},\gamma_{\tau }\right)-\sum_{r\in R}c_{r}\left(s_{d}\right)p_{c}, \end{array}$$ (6)

where *c*_*r*_(**s**_**d**_) is the size of the cache storage required to cache contents of the size **s**_**d**_.

## Joint deep learning and auction for congestion-aware caching (DeepAuC)

We present our deep learning model for predicting transit traffic in Subsection. The output of our prediction helps the ANP to identify the future volume of the transit traffic and the contents that have high demands over the transit links, in light of which, the ISP makes cache decisions. Because paid-contents require payments to be downloaded and cached, in Subsection, we describe in detail our auction model, which helps the ANP to obtain paid-content at an optimal price.

### Deep learning model

To prevent congestion on transit links, we propose a deep learning model that helps the ISP to record the transit traffic volume, learn the transit traffic characteristics, and predict the traffic volume that needs to be sent over the transit link(s). This helps the ISP to identify the contents that have high traffic volume/demands over the transit link(s). To reduce the traffic volume on the transit links, the ANP downloads and caches such identified contents.

In our prediction model, we learn the transit traffic characteristics from the recorded network traffic in the form of a traffic matrix (TM) denoted *X*^*τ*^. We consider *X*^*τ*^ to have dimensions *K* × *K* × *T* × *D*, where in the *X*^*τ*^ entries, we use $x_{kl}^{\tau }$ to represent the traffic volume that goes through the transit link τ∈T from the outgoing face k∈K of the GR of the ANP to the incoming face l∈K of the GR of the TNP. We transform $x_{kl}^{\tau }$ to $x_{klt}^{\tau }$ by adding *t* as the time dimension. Furthermore, to identify the contents that pass through the transit link τ∈T, we transform $x_{klt}^{\tau }$ to $x_{kltd}^{\tau }$ by adding *d* as the content dimension. Then, we use *X*^*τ*^ to predict the future TM denoted *Y*^*τ*^ using the LSTM recurrent neural network described below.

#### LSTM network overview

The LSTM [29] maps the input ***X***^*t*^ to the output ***Y***^*t*^ by computing the following function:

$$\begin{array}{c} \begin{array}{c} f_{t}=\sigma \left(W_{f}⊙\left[y_{t-1},x_{t}\right]+b_{f}\right), \end{array} \end{array}$$ (7)

where *σ* is the sigmoid function, *f*_*t*_ is the forget gate, *y*_*t*−1_ is the input from the previous layer, i.e., the output of the previous layer, and *b*_*f*_ is the bias. The output of the sigmoid function is a number between 0 (which means forget this value) and 1 (which means keep this value), which is used to decide the value to add to the cell state *c*_*t*−1_. To decide which information to store in the LSTM cell, two functions are used, where the sigmoid function at the input gate *i*_*t*_ is used to decide which value to update, while the tanh function is used to decide the new candidate values to add to the cell state denoted $c_{t}^{\prime }$:

$$\begin{array}{c} \begin{array}{c} i_{t}=\sigma \left(W_{i}⊙\left[y_{t-1},x_{t}\right]+b_{i}\right), \end{array} \end{array}$$ (8)

$$\begin{array}{c} \begin{array}{c} c_{t}^{\prime }=\text{tanh}\left(W_{c}⊙\left[y_{t-1},x_{t}\right]+b_{c}\right). \end{array} \end{array}$$ (9)

After computing the above functions, the next step is to update the cell state information from cell state *c*_*t*−1_ to *c*_*t*_ by computing the following function:

$$\begin{array}{c} \begin{array}{c} c_{t}=f_{t}*c_{t-1}+i_{t}*c_{t}^{\prime }. \end{array} \end{array}$$ (10)

Finally, the last step is to decide the output, where the sigmoid function is used to decide which part of the cell state to output and the tanh function is used to fit the output value in the range between 0 and 1:

$$\begin{array}{c} \begin{array}{c} o_{t}=\sigma \left(W_{o}⊙\left[y_{t-1},x_{t}\right]+b_{0}\right), \end{array} \end{array}$$ (11)

$$\begin{array}{c} \begin{array}{c} y_{t}=o_{t}*\text{tanh}\left(c_{t}\right). \end{array} \end{array}$$ (12)

#### Feeding the traffic matrix into the LSTM network

Our aim is to prevent congestion before it happens on transit links by predicting the TM. We use ***X***^*t*^ to denote a vector of the TM *X*^*τ*^ at the time slot *t*. We transform TM *X*^*τ*^ to ***X***^*t*^ by concatenating each row from top to bottom. Here, we consider TM prediction by solving ***X***^*t*^ using the historical measured transit traffic ***X***^*t*−1^, ***X***^*t*−2^, …***X***^*t*−*T*^, which requires continuous feeding and learning vectors of the traffic. However, to minimize the complexity of the computation, we adopt the learning window described in Ref. [28]. The learning window is used to fix the number of time slots *T* of the measured transit traffic vectors that need to be fed into the LSTM network to predict TM ***Y***^*t*^, where we use ***Y***^*t*^ to denote a vector of the predicted TM *Y*^*τ*^ at the time slot *t*.

#### Prediction methodology

As a prediction methodology, the measured transit traffic data are divided into two datasets. The first dataset (a training dataset) is used during the training phase to make the model and to identify the model parameters. Furthermore, the second dataset (the validation or testing dataset) is used to evaluate the performance of the prediction model. The workflow of our prediction model is summarized as follows.

- The prediction phase: At each time slot *t*, the model calculates the output ***Y***^*t*^ from the input ***X***^*t*^. Here, the inputs are the previous observations of the transit traffic ***X***^*t*−1^, ***X***^*t*−2^, …***X***^*t*−*T*^.
- The training phase: In this phase, we identify the model parameters by feeding the training data into the LSTM model and adjusting learning hyperparameters.
- The validation phase: After training and prediction, the final phase is validation. In this phase, we feed a validation dataset into the LSTM model and evaluate the accuracy of our prediction using the Mean Square Error (MSE) function described below:
  $$\begin{array}{c} \text{MSE}=\frac{\sum_{t=1}^{T}{∥x_{kltd}^{\tau }-y_{kltd}^{\tau }∥}^{2}}{T}, \end{array}$$ (13)
  where $x_{kltd}^{\tau }$ is the ground truth value and $y_{kltd}^{\tau }$ is the predicted value.

To prevent congestion on each transit link τ∈T, the predicted traffic volume for each transit link τ∈T needs to satisfy the following constraint:

$$\begin{array}{c} \rho_{\tau }^{\prime }=\sum_{u\in U(\tau )}\sum_{d\in D}s_{ud}y_{kltd}^{\tau }\left(1-h_{ud}\right)\leq C_{\tau }, \end{array}$$ (14)

where $\rho_{\tau }^{\prime }$ is the predicted traffic volume on each transit link *τ*. If the ANP network cannot satisfy Constraint in (14), congestion will occur on link *τ*.

### Auction model for congestion-aware caching

**Fig 2:**
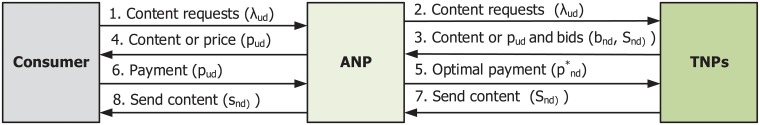
Auction workflow for congestion-aware caching.

When the ANP network cannot satisfy Constraint (14), to prevent congestion and reduce the traffic volume on the transit links, the ANP downloads and caches contents that have high predicted traffic volume. However, downloading and caching paid-contents require payments from the ANP to the TNPs. Therefore, to obtain the paid-content at an optimal price that minimizes total payment of the ANP, we propose an auction approach. The workflow of the proposed auction model is shown in Fig 2 and works as described below.

- *Step 1*: Each consumer u∈U(i) requests each content *d* by sending an Interest packet λ_*ud*_ via an internal link i∈I.
- *Step 2*: For the requested content *d* that is not cached inside the ANP network, when the ANP network cannot satisfy Constraint (14) and the requested content *d* has high predicted traffic volume $y_{kltd}^{\tau }$ and needs to use the transit link *τ*. Then, the ANP requests that content by sending a request λ_*ud*_ to all transit links.
- *Step 3*: For free content, any TNP, which has the requested content in its cache storage, returns the content to the ANP. However, for paid-content, each TNP *n* returns a bid (*b*_*nd*_, *s*_*nd*_) and *p*_*ud*_, where *b*_*nd*_ is the content price, *s*_*nd*_ is the size of the content *d* submitted by TNP *n*, and *p*_*ud*_ is the consumer price.
- *Step 4*: The ANP delivers free content to the consumer for free and the workflow ends. However, for paid-content, the workflow continues by returning the content price *p*_*ud*_ to the consumer. Simultaneously, the ANP takes all the bids from the TNPs and performs an evaluation of the bids via reverse auction to select the winner and calculate the optimal price $p_{nd}^{*}$ of each content *d*.
- *Step 5*: The ANP informs the winner and pays the optimal price $p_{nd}^{*}$ to each winning TNP *n* for each content *d* that needs to be cached.
- *Step 6*: For paid-content *d*, each consumer u∈U(i) needs to pay the content price $p_{ud}\geq p_{nd}^{*}$ as the fixed price from the TNP. In other words, each consumer u∈U(i) needs to pay *p*_*ud*_ for each paid-content d∈D, whether or not the content *d* is cached inside the ANP network.
- *Step 7*: The ANP caches the selected and delivered contents in its cache storage to serve future requests from consumers of the same contents.
- *Step 8*: Finally, the ANP delivers the paid-content to its consumer.

#### Auction model

The ANP needs to pay the price *p*_*nd*_ to cache the paid content *d*, which is predicted to come from a highly congested transit link by our prediction model. We use Reverse Auction Congestion-Aware Caching (RACAC) to obtain each paid content *d* at the lowest price. We chose reverse auction over other auction mechanisms because reverse auction allows the ANP as a buyer to request bids from multiple TNPs (sellers) and select the bidding value that minimizes its payments. In addition, RACAC allows TNPs to bid the prices at which they are willing to sell their contents. Here, free contents are not considered in RACAC.

**Definition 0.1** (RACAC). In our RACAC, we consider that the ANP needs to obtain the high predicted content *d*, which requires using the transit link from the TNPs at the lowest content price. The ANP floods Interest packet as demand to the transit link(s). Upon receiving an Interest packet, any TNP n∈N that has the requested content submits a bid *b*_*nd*_. Then, the ANP chooses the bidding value that minimizes its payment.

In the RACAC, each TNP *n* has its own valuation denoted *G*_*n*_(*s*_*nd*_) for the content *d*, where *G*_*n*_(*s*_*nd*_) is given by:

$$\begin{array}{c} G_{n}\left(s_{nd}\right)=\{\begin{array}{c} g_{nd},\;\text{if}\;\text{TNP}\;n\;\text{participates}\;\text{in}\;\text{RACAC},\\ +\infty ,\;\text{otherwise,} \end{array} \end{array}$$ (15)

where *g*_*nd*_ is a true valuation of TNP *n* for the paid-content *d*. However, when TNP *n* does not participate in RACAC, it is assigned a valuation of infinity. In addition, we use *G*_*N*_ to denote the minimum valuation of all the TNPs, where *G*_*N*_ is expressed as follows:

$$\begin{array}{c} \begin{array}{c} G_{N}=\underset{s_{d}^{*}}{\text{min}}\sum_{n\in N}^{}f_{d(\tau )}b_{nd}\left(s_{nd}^{*}\right),\forall d\;\in D, \end{array} \end{array}$$ (16)

where $s_{d}^{*}=\left\{s_{nd}^{*}\right\}$ is a vector containing the highest predicted contents that need to be cached to prevent congestion on the transit links.

To analyze RACAC and determine the optimal price $p_{nd}^{*}$ for the content *d*, we use the Vickrey–Clarke–Groves (VCG) approach [32]. We chose VCG over other approaches because VCG is simple and easy to implement. In addition, VCG guarantees a truthful outcome and a social-optimal solution. Therefore, based on the VCG approach, each TNP n∈N needs to compensate for any harm that it (TNP *n*) may cause to other TNPs by participating in RACAC. We calculate the total minimum valuation *G*_−*n*_ of the TNPs other than TNP *n* as follows:

$$\begin{array}{c} G_{-n}=\underset{s_{d}^{*}\prime }{\text{min}}\sum_{m\in N\backslash \{n\}}^{}f_{d(\tau )}b_{md}\left(r_{md}^{\prime }\right),\forall d\;\in D, \end{array}$$ (17)

where $s_{d}^{*}\prime$ = $\left\{s_{md}^{*}\right\}$ is a vector containing the highest predicted contents that need to be cached to prevent congestion on the transit links when TNP *n* does not participate in RACAC. Therefore, the social-optimal price $p_{nd}^{*}$ for the content *d* that the ANP needs to pay the winner is given by

$$\begin{array}{c} \begin{array}{c} p_{nd}^{*}=G_{-n}-\underset{s_{d}^{*}}{\text{min}}\sum_{m\neq n}^{}f_{d(\tau )}b_{md}\left(s_{md}^{*}\right),\forall n\;\in W, \end{array} \end{array}$$ (18)

where W⊂N is the set of winners. To make sure that RACAC guarantees a truthful outcome and a social-optimal solution, below, we define and prove the essential properties of RACAC.

**Definition 0.2** (Truthfulness of RACAC). We consider RACAC to be truthful if every TNP n∈N chooses to bid its true valuation (*b*_*nd*_ = *g*_*nd*_) over other possible bidding values (*b*_*nd*_ ≠ *g*_*nd*_). RACAC is truthful if it guarantees that each bidding value *b*_*nd*_ = *g*_*nd*_ of TNP n∈N maximizes the utility of TNP over the other possible bidding values (*b*_*nd*_ ≠ *g*_*nd*_) for the content *d*.

**Theorem 0.1**. *Truthfulness of RACAC*.

*Prrof*. Here, to prove the truthfulness of RACAC, we use critical payment and the monotonicity conditions described in Ref. [33].

To satisfy the monotonicity condition, we assume that a TNP n∈N sends to auction two bidding values $b_{nd}^{\prime }$ and *b*_*nd*_ for the content *d*, where $b_{nd}<b_{nd}^{\prime }$. RACAC selects the TNP n∈N whose bidding value minimizes Eq (16) as the winner. With the increasing order of the bidding values, if TNP n∈N wins RACAC by bidding $b_{nd}^{\prime }$, and because $b_{nd}<b_{nd}^{\prime }$, TNP n∈N will also win the RACAC for any bidding value $b_{nd}<b_{nd}^{\prime }$.

To satisfy critical payment, each TNP n∈N with a minimum bidding value smaller than that of the other TNPs always wins RACAC and is paid $p_{nd}^{*}$ guaranteeing non-negative utility ($p_{nd}^{*}\geq g_{nd}$), where $p_{nd}^{*}$ is considered to be the critical payment. Therefore, when considering both the monotonicity and critical payment conditions, for each TNP n∈N, submitting a bidding value $\left(b_{nd}^{\prime }\neq g_{nd}\right)$ that diverges from its true valuation (*g*_*nd*_ = *b*_*nd*_) will not be beneficial and therefore the TNP will not be better off. In conclusion, RACAC is truthful; therefore, it guarantees truthful bidding (*b*_*nd*_ = *g*_*nd*_) to be a dominant strategy for every TNP n∈N.

**Definition 0.3** (The utility of the TNP for paid-content). For the content d∈D, we assume that each TNP n∈N submits a bid *b*_*nd*_. If TNP n∈N wins the auction, it will receive $p_{nd}^{*}$ as payment. Otherwise, TNP n∈N receives zero payment if it has lost the auction. The utility *U*_*n*_ of any TNP n∈N is expressed as follows:

$$\begin{array}{c} U_{n}=\{\begin{array}{c} p_{nd}^{*}-g_{nd},\;\text{if}\;\text{TNP}\;n\;\in W\text{,}\\ 0,\;\text{otherwise}. \end{array} \end{array}$$ (19)

From Eq (19), we can define the individual rationality as follows.

**Definition 0.4** (Individual rationality). We consider RACAC to be individually rational if and only if every TNP n∈N does not have negative utility. In other words, $p_{nd}^{*}\geq g_{nd}$ and *U*_*n*_ ≥ 0 for every TNP n∈N.

**Theorem 0.2**. *Individual rationality*.

*Proof*. Here, to prove that RACAC is individually rational, we use the individually rational property defined in Ref. [33]. Based on Theorem 0.1, RACAC guarantees individual rationality, because RACAC selects the TNP n∈N with the minimum bidding value as the winner, where the winner n∈W receives $p_{nd}^{*}$ as payment, which is considered to be the critical payment. Therefore, regardless of the bidding values of the other TNPs, the loser ANP receives $p_{nd}^{*}=0$, while the winner TNP receives $p_{nd}^{*}\geq b_{nd}$ as payment, which guarantees that *U*_*n*_ ≥ 0 (Eq 19). In other words, no TNP receives negative utility.

### Problem formulation

We formulated a joint deep learning and auction-based approach for congestion-aware caching as an optimization problem that minimizes the content caching cost. Therefore, the RACAC Cost Minimization (RACAC-CM) problem is given by

$$\underset{f_{d}}{\text{min}}\sum_{n\in N}f_{d(\tau )}^{n}g_{nd}\left(s_{nd}\right)$$ (20)

subject to:

$$\sum_{n\in N}\sum_{d\in D}f_{d(\tau )}^{n}\rho_{\tau }^{\prime }\leq C_{\tau },\;\forall \tau \in T,$$ (20a)

$$\sum_{n\in N}\sum_{d\in D}f_{d(\tau )}^{n}\leq 1,$$ (20b)

$$\sum_{n\in N}\sum_{d\in D}f_{d(\tau )}^{n}s_{nd}\geq \sum_{d\in D}s_{ud},\;\forall \tau \in T,$$ (20c)

$$\sum_{n\in N}\sum_{d\in D}f_{d(\tau )}^{n}g_{nd}<\sum_{d\in D}p_{nd},$$ (20d)

$$\sum_{n\in N}\sum_{d\in D}f_{d(\tau )}^{n}\beta_{d}\geq \sum_{d\in D}\theta_{d},$$ (20e)

$$\sum_{n\in N}\sum_{d\in D}f_{d(\tau )}^{n}s_{nd}\leq \sum_{r\in R}c_{r},\;\forall r\in R,$$ (20f)

$$f_{d(\tau )}^{n}\in \left\{0,1\right\},\;g_{nd}\in \left[0,+\infty \right).$$ (20g)

*Constraints*: Constraint (20a) ensures that the volume of the transit traffic at each transit link τ∈T is less than or equal to *c*_*τ*_. We use Constraint (20b) to ensure that each paid-content *d* is delivered by one TNP n∈W. Further, to ensure that each content size *s*_*nd*_ of TNP n∈W is greater than or equal to the content size *s*_*ud*_ needed by the customer of the ANP, we use Constraint (20c). Constraint (20d) (*b*_*nd*_ < *p*_*nd*_) ensures individual rationality. Constraint (20e) guarantees that the content that needs to be cached enables the ANP to reduce the transit traffic volume and network latency. Constraint in (20f) ensures that the paid-contents that need to be kept in cache storage are less than or equal to the cache capacity *c*_*r*_ of the ANP.

*Variables*: We use $f_{d}=\left\{f_{d(\tau )}^{n}\right\},\forall n\in N,d\in D$ as a vector of the decision variables, and $f_{d(\tau )}^{n}$ is expressed as follows:

$$\begin{array}{c} f_{d(\tau )}^{n}=\{\begin{array}{c} 1,\;\text{if}\;\text{bidder}\;n\in W\;\text{and}\;f_{d(\tau )}=1,\forall d\;\in D,\\ 0,\;\text{otherwise}. \end{array} \end{array}$$ (21)

Therefore, *g*_*nd*_ is private information and each TNP n∈N does not share *g*_*nd*_ with the ANP. We transform the RACAC-CM problem into a RACAC Payment Minimization (RACAC-PM) problem by replacing *g*_*nd*_ with *b*_*nd*_ (*b*_*nd*_ is known by the ANP), where the RACAC-PM problem is given by

$$\begin{array}{c} \begin{array}{c} \underset{f_{d}}{\text{min}}\sum_{n\in N}f_{d(\tau )}^{n}b_{nd}\left(s_{nd}\right). \end{array} \end{array}$$ (22)

Hereafter, we focus on solving Eq (22), where Eq (22) has the same constraints and variables as Eq (20).

**Theorem 0.3**. *The NP-hard hardness of the RACAC-PM problem*, Eq (22).

*Proof*. To prove that the RACAC-PM problem, Eq (22), is NP-hard, we formulate it in its conjunctive normal form [34] by considering **b**_**d**_ to be a vector of the bidding values in the RACAC-PM. Therefore, B = $B_{1}\cap B_{2}\cap \ldots \cap B_{m}$ is the conjunctive normal form, where $B_{m}$ is a set of bidding values from the TNPs. Further, each $B_{m}$ is defined as a disjunction of *m* bids {*b*_1*d*_} ∪ … ∪ {*b*_*md*_}. We assume that each bid has no negative bidding value *b*_*md*_ for the content *d*. Further, for every value *b*_*md*_, we can transform B into an arithmetic form *χ* using $f_{d(\tau )}^{m}$ as described in Eq (21). Then, by interchanging ∩ with * and ∪ with +, *χ* becomes an NP-hard problem, because *χ* ≥ 1 for $0\leq f_{d(\tau )}^{m}\leq 1$.

### Winner and price determination

**Algorithm 1** Winner Determination.

1: **Input**: **C**, ***ρ***, **c**, **b**_**d**_, **p**_**d**_, **s**_**d**_, N, $\beta_{d}$, $\theta_{d}$;

2: **Output**: **f**_**d**_, W, $s_{d}^{*}$, $s_{d}^{*}\prime$, *G*_*N*_, N, $N^{\prime }$;

3: Initialize: **f**_**d**_ ← (0, …, 0), W←∅, *G*_*N*_ ← 0,

  ***R*** ← (0, …, 0), *count*[*s*_*nd*_] ← 0;

4: **for all b**_**d**_ < **p**_**d**_, **s**_**d**_ ≠ (0, …, 0), and *β*_*d*_ ≥ *θ*_*d*_ **do**

5:  **for all** *s*_*nd*_ ∈ **s**_**d**_ **do**

6:   *count*[*s*_*nd*_] = *count*[*s*_*nd*_] + 1;

7:  **end for**

8:  ***R*** ← {*s*_*nd*_, (*count*[*s*_*nd*_])};

9:  In ***R***, find content size *s*_*nd*_ with maximum *count*[*s*_*nd*_]: *s*_*ud*_ ← *s*_*nd*_;

10:  **while** ***ρ*** ≤ **C**, *β*_*d*_ ≥ *θ*_*d*_, $s_{d}^{*}\leq c$, *b*_*nd*_ ≥ 0, and *s*_*nd*_ ≥ *s*_*ud*_ **do**

11:   $s_{d}^{*}\leftarrow s_{nd};$

12:   Sort bidding values **b**_**d**_ in ascending order;

13:   Find a TNP n∈N with minimum bid value: *n* = *min*(**b**_**d**_);

14:   *G*_*N*_ ← *G*_*N*_ + *b*_*nd*_(*s*_*nd*_);

15:   $N^{\prime }\leftarrow N\backslash \left\{n\right\};$

    $s_{d}^{*}\prime \leftarrow s_{d}^{*}\backslash b_{nd};$

16:   W←W∪{n};

17:   $f_{d(\tau )}^{n}\leftarrow 1;$

18:   $f_{d}\leftarrow f_{d(\tau )}^{n};$

19:  **end while**

20: **end for**

21: **Return**: **f**_**d**_, W, $s_{d}^{*}$, $s_{d}^{*}\prime$, *G*_*N*_, N, $N^{\prime }$.

The formulated problem in Eq (22) is NP-hard, which is proved in Theorem 0.3. Even the NP-hard problem can be solved using exhaustive search, when the size of the problem becomes large, the exhaustive search requires high computational resources. Therefore, as described in [35], it is possible to analyze and design a faster algorithm than exhaustive search for solving specific NP-hard problem. For this reason, to solve Eq (22), we propose two algorithms: Winner Determination (Algorithm 1) and Price Determination (Algorithm 2). We use the VCG approach [32] to design both the algorithms. In addition, in the formulated problem, we assume that the ANP has sufficient bandwidth for internal links. Therefore, in both algorithms, we focus on the transit links that have limited bandwidth capacities.

In our proposal, we use Algorithm 1 to determine the TNP n∈N that can deliver the requested content *d* at a minimum price/ bidding value. For input, we use a vector of transit links having capacities **C**, a vector of predicted volumes of transit traffic ***ρ***, a vector of cache capacities **c**, a set of TNP N, a vector of bidding values **b**_**d**_, a vector of fixed content prices **p**_**d**_ that consumer pays for each content *d*, a vector of content sizes **s**_**d**_, the delay tolerance threshold *θ*_*d*_ > 0, and the demand delay product *β*_*d*_. Algorithm 1 performs initialization on line 3. In addition, to identify the content size (lines 4−9), we use support count, as described by Ref. [11]. The content size, which has high value of support count, is treated by the ANP as the baseline content size needed and most often requested by its consumers. Support count helps the ANP to buy and cache the most requested contents. Therefore, the ANP sells the cached content to its consumers. The content that has a high support count value needs to have high QoS (minimized delay) because it allows the ANP to increase its profits. In addition, caching content that has a high support count value helps the ANP to save on the bandwidth cost for transit traffic.

**Algorithm 2** Price Determination.

1: **Input**: **f**_**d**_, W, $s_{d}^{*}$, *G*_*N*_, **b**_**d**_, $N^{\prime }$, $s_{d}^{*}\prime$, N;

2: **Output**: $p_{d}^{*}$

3: Initialize: $p_{d}^{*}\leftarrow \left(0,\ldots ,0\right)$, *G*_−*n*_ ← (0, …, 0), $W^{\prime }\leftarrow \emptyset$;

4: **while** *b*_*md*_ ≥ 0, $m\in N^{\prime }$, and $s_{md}^{*}\in s_{d}^{*}\prime$ **do**

5:  Find a TNP $m\in N^{\prime }\cup W\backslash \left\{n\right\}$ that has a smallest bid value: *m* = *min*(**b**_**d**_);

6:  ***G***_−***n***_ ← ***G***_−***n***_ + *b*_*md*_;

7:  $W^{\prime }\leftarrow W^{\prime }\cup \left\{m\right\};$

8:  $p_{nd}^{*}=G_{-n}-\underset{s_{d}^{*}}{\text{min}}\sum_{m\neq n}^{}b_{md}\left(s_{md}^{*}\right))$;

9:  $p_{d}^{*}\leftarrow p_{nd}^{*}$;

10: **end while**

11: **Return**: $p_{d}^{*}$.

For the content size *s*_*nd*_ ≥ *s*_*ud*_ (where *s*_*ud*_ is the baseline content size) and *β*_*d*_ ≥ *θ*_*d*_, Algorithm 1 performs the winner determination computation on lines 10−19. However, when *s*_*nd*_ < *s*_*ud*_ for a content *d*, there is no motivation for the ANP to cache it because the content *d* is not frequently requested by its consumers. Further, without violating the link and cache capacity constraints, Algorithm 1 performs iterations until the TNP n∈N that has a minimum bidding value wins the RACAC (line 13) for each content *d* of size *s*_*nd*_ ≥ *s*_*ud*_. Moreover, from line 14 to line 16, Algorithm 1 calculates the total valuation and appends the TNP *n* that has the minimum bidding value to the set of winner W, excludes the winner n∈W to the set N of remaining bidders, and creates a new set $N^{\prime }$ of bidders on line 17. Finally, on line 21, the algorithm returns the vector **f**_**d**_ of the decision variables and the set W of the winning TNPs as the output.

After the winner determination in Algorithm 1, we need to calculate the optimum price $p_{nd}^{*}$ that the ANP has to pay to each winning TNP n∈W for each paid content *d*. We use Algorithm 2 to calculate the optimum price $p_{nd}^{*}$ for each paid content *d*. For input, we use the sets of the TNPs (N and $N^{\prime }$), **f**_**d**_, *G*_*N*_, **b**_**d**_, $s_{d}^{*}$, W, and $s_{d}^{*}\prime$. Algorithm 2 performs initialization on line 3. From line 4 to line 10, when *b*_*md*_ ≥ 0 and $s_{md}^{*}\in s_{d}^{*}\prime$, Algorithm 2 performs iterations to compute the optimal price $p_{nd}^{*}$ that the ANP needs to pay to each TNP n∈W for caching each content *d*. Finally, on line 11, Algorithm 2 returns the vector of the optimal prices $p_{d}^{*}$ as the output. Here, the ANP needs to pay $p_{d}^{*}$ to the winning TNPs for caching the paid-contents.

For the computational complexity of both proposed algorithms (Algorithms 1 and 2), in light of our previous work [11], we can make the following remark.

*Remark*. The computational complexity of the proposed Algorithms 1 and 2 is *O*(*n*^3^).

## Performance evaluation

In this section, we present and discuss the performance evaluation of our proposed DeepAuC. We use ndnSIM, which is an ns-3 based simulator for NDN [36] [37]. In addition, we use Keras with TensorFlow on the back-end [38] for deep learning and pandas [39] for the data analysis. We installed these tools in PC with Intel(R) Core(TM) i5-4670 CPU 3.40 GHz and 8GB of RAM. The simulation codes are available online [40].

### Simulation setup

For the simulation setup, we use the GEANT topology as a realistic network topology. The GEANT network interconnects research centers and universities in Europe [41]. To map GEANT topology with the ANP and TNPs for auction, we extend GEANT topology with 20 consumers and one CP. In the topology, we have 23 GRs, where we connected 20 consumers with one GR for forming the ANP network. The GR of the ANP is connected to the remaining 22 GRs, where we assume that 22 GRs are the GRs of the TNPs. In other words, in our action, we have one ANP and 22 TNPs. This mapping is clearly visible in [40] (GEANT2_deep_cache_topology.txt). In the implemented GEANT topology, the link capacities between the consumer nodes and GRs are in the range of 6−10 Mbps, while the link capacity between each two GRs is 10 Mbps. For each link, we use a queue of 100 packets and the transmission delay equals *β*_*d*_ = 10 ms and *θ*_*d*_ = 9 ms. In addition, each consumer u∈U generates λ_*ud*_ = 300 interest packets per second. We use three types of contents: video, news, and music files.

To get the dataset, in NDN, we use GEANT network topology described in [40] (filename: GEANT2_deep_cache_topology.txt). We collected network traffic data generated by 20 consumers for a period of 4 hours for both the training and testing purposes. In addition, in both training and testing datasets, at each time slot (0.5 seconds), we have content names, incoming and outgoing faces, and content size in terms of kilobyte as features. At the GR of the ANP, outgoing faces record the traffic going outside the ANP network, while incoming faces record the traffic coming inside the ANP network. We used the training dataset to make the LSTM model and identify the LSTM model parameters. In addition, we used the testing dataset to test the performance of our prediction model. The training and testing datasets are available in [40] (training_dataset3.csv and testing_dataset3.csv), while the source code for collecting the dataset is available with the filename GEANT2_deep_cache.cpp [40].

For prediction purpose, we fed the collected traffic data into our LSTM model to predict future volume of the transit traffic that needs to pass through the transit links. To determine the LSTM model, we repeatedly fine-tuned hyperparameters such as a number of layers and neurons, dropout, number of epochs, and learning rate until we obtain better performance. Such approach is practical in deep learning for finding the appropriate model structure [42]. Therefore, our LSTM model reached to the good performance when it had one input layer, four hidden layers, and one output layer. In our LSTM model, we used dropout regulation technique to prevent overfitting, where the dropout parameter was set to 0.2. Each layer had 128 units/neurons except for the output layer which had one neuron. In addition, we used the Adam optimizer [43] to update the model and MSE as the error function. The Adam optimizer is an extension of stochastic gradient descent for updating deep learning network weights, where the weights update depends on the training dataset. Further, we sorted the predicted traffic at each transit link in descending order and deployed cache storage on the GRs connected to the transit links that had a high predicted traffic volume. We used cache storage in the range from *c*_*r*_ = 10, 000 to *c*_*r*_ = 1, 000, 000 data packets, where each packet had a payload of size 1024 bytes. We assumed a transit fee *γ*_*τ*_ = 0.63 USD per 1 Mbps and a cache storage price of *p*_*c*_ = 0.003625 USD per 1 Mb. In addition, each consumer u∈U pays 50 USD as a monthly access bandwidth fee per 1 Gb [11].

For the proposed auction model, to cache the contents that have high predicted demands, we consider 22 TNPs that can provide contents to the ANP for caching on payment (paid-content) or without payment (free content); however, we only focus on the paid-contents. Further, the bidding value *b*_*nd*_ for each content *d* submitted by each bidder n∈N is generated randomly within the range from *b*_*nd*_ = 2 USD to *b*_*nd*_ = 8 USD, while the consumer price *p*_*ud*_ is generated randomly within the range from *p*_*ud*_ = 6 USD to *p*_*ud*_ = 12 USD. We obtain the ANP, TNP, and CP profits for the paid-contents using the content distribution and reselling approach introduced by Kreuzer *et. al*. [44]. In this approach, for each additional time the cached content *d* is sold, each ANP and TNP n∈W receive 15% of the *p*_*ud*_. In other words, the ANP pays $p_{nd}^{*}$ for caching and the first sale of each content *d*. Then, from the second sale of the cached content *d*, the ANP forwards 85% of the *p*_*ud*_ to the TNP n∈W for each content *d* sold. In addition, each TNP n∈W receives 15% of the *p*_*ud*_ as the content distribution price and forwards 70% of the *p*_*ud*_ to the CP.

In the performance evaluation, we did not include all components of our proposal in ndnSIM because we use different tools (Keras with TensorFlow on the back-end and pandas) and languages (Python, Julia, and C++), which require time and human resources to redesign/extend the ndnSIM simulator. However, to reproduce our proposal and results, we summarize the connection between all components of our proposal and all the procedures for utilizing our source code available in [40] as follows:

- First, in ndnSIM, we used GEANT2_deep_cache.cpp and GEANT2_deep_cache_topology.txt to collect dataset. We run GEANT2_deep_cache.cpp two times (each time, for a period of 4 hours) for collecting training and testing datasets. Then, we save the collected datasets in rate_trace_GEANT2_myproposal.txt files.
- Second, we perform data cleaning by removing unwanted features such as face description and type. Then, we save the outputs in .csv format (traffic_dataset_4_train3.csv, traffic_dataset_4_test3.csv).
- Third, we map the collected datasets to the topology to obtain the connection between nodes, bandwidth, metric, delay, and queue. Then, we identify the names of the contents that passed in both outgoing and incoming faces of the transit links. The Python source codes for data preparation are available in Data_Preparation3.py. We save the output of data preparation in .csv format (training_dataset3.csv and testing_dataset3.csv).
- Fourth, we feed the cleaned and prepared datasets in LSTM model, where we use training_dataset3.csv for the training dataset and testing_dataset3.csv for the testing dataset. Then, we predict the future demands for contents need to pass through the transit links, where the output of our prediction is saved in network_traffic_prediction3.csv. The python source codes of our prediction are available in Prediction_Traffic3.py [40].
- Fifth, because we use 20 time slots for the window size or the loopback value which cause our prediction approach to start having the predicted transit traffic from 21*st* time slot, we removed the rows that have missing the predicted transit traffic (the first 20 time slots). Then, we save the final result in network_traffic_prediction3.csv [40].
- Sixth, we feed the prediction output (network_traffic_prediction3.csv) in our auction codes and randomly generate the bidding values for purchasing the contents that have high predicted future demands and need to pass through the transit links. The Julia source codes for our auction are available in gurobi_vcg_01.jl [40].
- Finally, to cache the purchased contents that have high predicted future demands and need to pass through the transit links, we update GEANT2_deep_cache.cpp and implement cache storage in ndnSIM to prevent congestion and minimize transit bandwidth consumption. The source codes for implementing cache storage in ndnSIM are available in GEANT2_deep_cache_update [40].

### Simulation results

All our data and source code used to generate the results have been made public for the betterment of the research community and are online and available in [40]. To collect the transit traffic data, we ran the ndnSIM simulation two times, the first time to collect training dataset (traffic_dataset_4_train3.csv [40]) and the second time to collect the testing dataset (traffic_dataset_4_test3.csv [40]).

**Fig 3:**
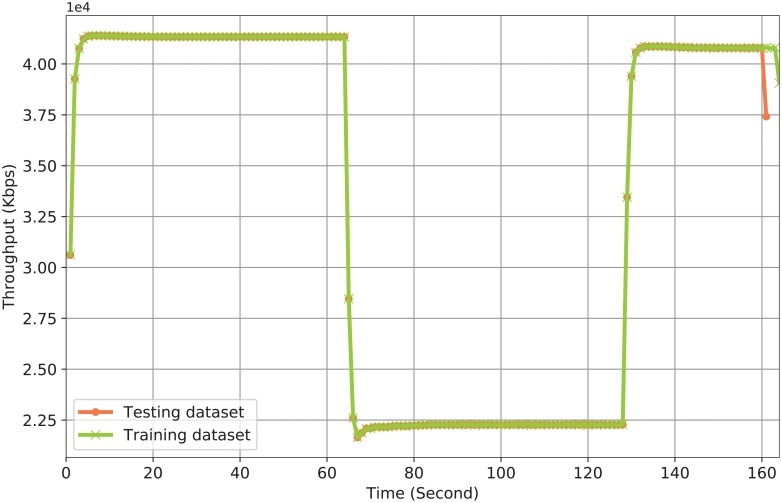
Collected network traffic data.

Fig 3 shows the ground truth of the network throughput. After analyzing the collected datasets, Fig 3 demonstrates that the packet drops due to congestion start after 64 second. The throughput degradation due to congestion from 64 second to 128 second causes not only packet drops but also packet retransmissions (the source code for generating this figure is available with the filename Data_Preparation3.py [40]).

**Fig 4:**
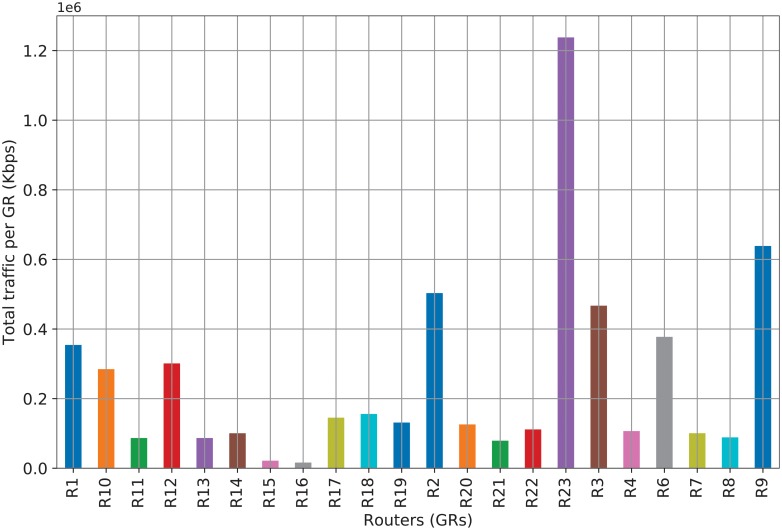
Total transit traffic volume per GR.

Further, to analyze the transit traffic of each GR k∈K, Fig 4 shows the volume of traffic that passes through each GR k∈K (the source code for generating this figure is available with the filename Prediction_Traffic3.py [40]). We can see in this figure that R23 has a large volume of transit traffic because R23 is the GR of the ANP, while the remaining routers are the GRs of the TNPs. In other words, in the ANP, all the consumer transit traffic needs to pass through R23. Hereafter, we use cache-enabled routers as GRs with CC functionalities.

**Fig 5:**
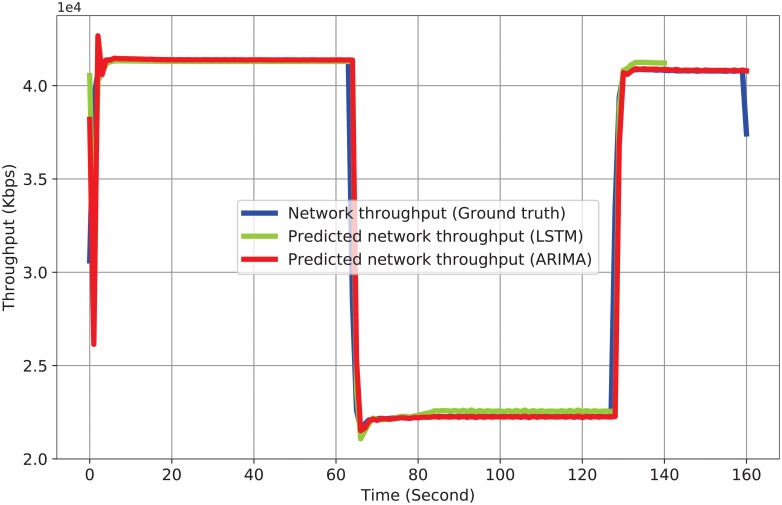
Network traffic prediction.

To prevent congestion and high delays on the transit links, where the transit links connect the GRs, we use both training and testing datasets to predict the future traffic volume. The collected transit traffic data over time are treated as time series data. To predict the transit traffic volume using the collected time series dataset and the LSTM model, we use 20 seconds for the window size or the loop_back value, where the loop_back value defines the number of previous time steps to be used as input. In addition, we use MinMaxScaler to shape the input values ranging from 0 to 1. This helps to prevent large margins between the collected input values. We use the training dataset to make an LSTM model and the testing dataset to test the model. After the prediction, we apply an inverse transformation to convert the transit traffic volume into a presentable format. Fig 5 shows the ground truth of the network throughput alongside the predicted network throughput. In this figure, we compare our LSTM model to the AutoRegressive Integrated Moving Average (ARIMA) model described in Ref. [45]. The simulation results show that the LSTM has a better performance than ARIMA (the source code for generating Fig 5 is available with filename Prediction_Traffic3.py [40]). LSTM performs well because it has a cyclic connection over time and memory that can be used to temporally store the internal state information of the prediction model.

**Fig 6:**
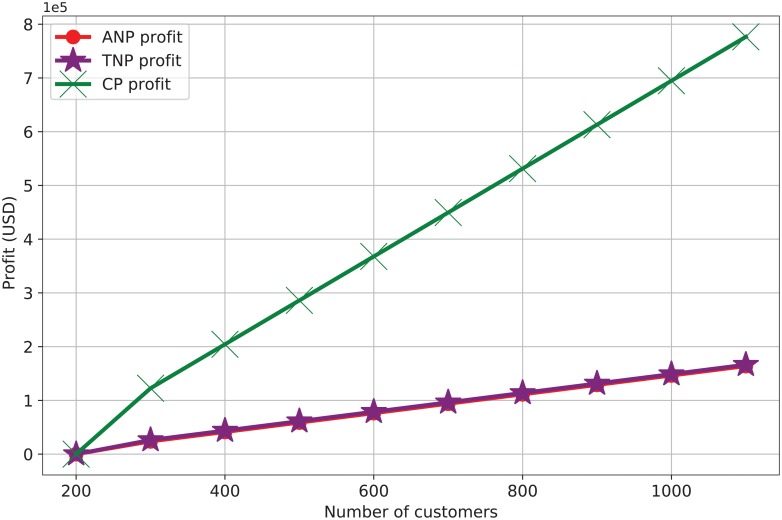
Caching profits (USD).

After predicting the transit traffic volume, we sort the transit demands for contents over the transit links in descending order. In this way, the ANP identifies the most requested contents over the transit links and obtains these contents via our auction model to prevent congestion and high delays (the source code for the auction model is available with filename gurobi_vcg_01.jl [40]). For the cached paid-contents, the simulation results in Fig 6 show an increase in the profits of the ANP, TNPs, and CP with increasing consumer demands. The ANP profit is based on selling contents, selling access bandwidth, and bandwidth savings due to transit traffic reduction. Further, the TNP profit is based on transit bandwidth payments from the ANP and content distribution payments. The CP generates profits by selling the paid-contents, and the ANP and TNPs need to forward to CP a certain percentage of the revenue earned by reselling the cache contents. Participating in selling contents helps both the ANP and TNP to increase their profits, and this can motivate both the TNPs and ANPs to adopt in-network caching of NDN architecture.

**Fig 7:**
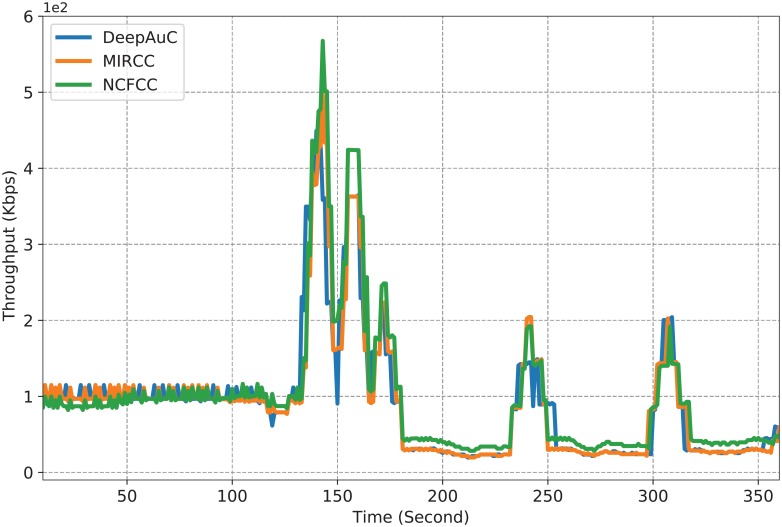
Comparison of the average network throughput.

After predicting and identifying the transit links that have high traffic volume, we deployed cache storage to reduce the volume of the transit traffic. The simulation results in Fig 7 show the average network throughput of our proposed method (DeepAuC) after deploying cache storage. In addition, we compared the throughput of our proposal to other similar congestion control approaches: NCFCC [20] and MIRCC [46]. The simulation results demonstrate that our proposal and MIRCC have nearly the same performance. In the beginning, both DeepAuC and MIRCC perform better than NCFCC. DeepAuC is based on transit traffic prediction. Conversely, MIRCC calculates the appropriate rate of traffic to send through each link. The weakness of NCFCC prior to 115 second compared to the DeepAuC and MIRCC approaches is that each node generates requests with an initial window size. Then, at the arrival of the data packet, the node increases the window size. However, on timeout or on reception of Reduce Sending Rate (RSR) message, the node needs to reduce the window size. Therefore, adjusting the window size based on the received data packet or timeout contributes to the slow start of NCFCC, causing the throughput of NCFCC to be very low for the first 115 seconds. When there is no timeout or RSR, NCFCC keeps increasing the window size, which contributes to its better performance after 115 second. The source code for our proposal after deploying cache storage is available with filename GEANT2_deep_cache_update.cpp [40]. The throughput data for generating Fig 7 and the source code are available with filename Géant_throughput_comparison.py [40].

**Fig 8:**
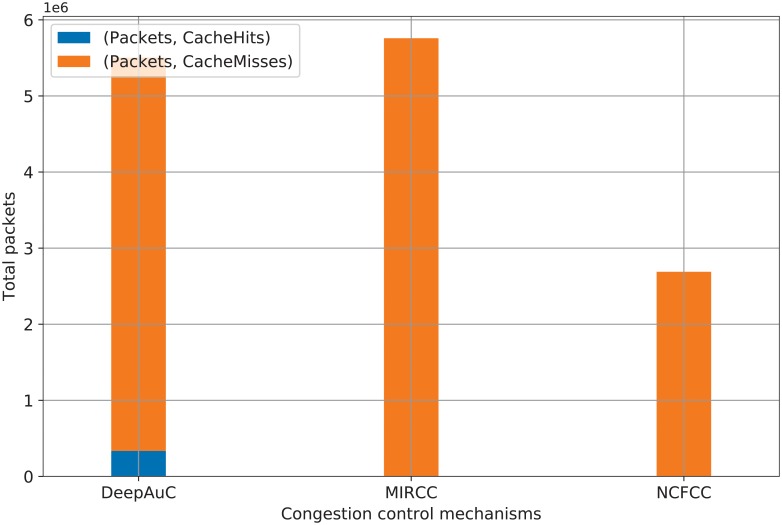
Comparison of cache hits and misses.

Because we measure the network throughput in terms of packets sent and received over time, we can trace the number of packets sent over the transit links using the different congestion control mechanisms being compared in this paper. The simulation results in Fig 8 show the total packets sent and the corresponding cache hits or misses. MIRCC and NCFCC have negligible cache hits because these mechanisms do not consider content caching as a way of preventing congestion. In other words, in case of cache misses, MIRCC and NCFCC have to send demands to upstream GRs, which contributes to their high network throughput in Fig 7. Conversely, DeepAuC prevents congestion by caching contents that have high predicted demands over the transit links. Fig 8 clearly demonstrates that DeepAuC has a remarkable number of cache hits and, therefore, reduces the number of packets that need to be sent to upstream GRs over transit links, i.e., the network throughput. The data and source code for generating Fig 8 are available with filenames cs-trace.csv and cs-trace.py, respectively [40].

**Fig 9:**
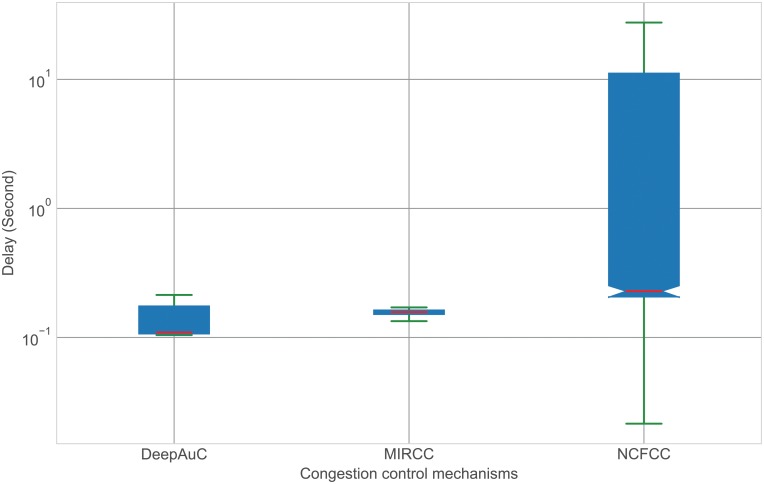
Comparison of the network delays.

Network congestion increases the number of dropped and retransmitted packets, which contributes to increases in the network delay. Therefore, to evaluate the performance of our proposal, we compare our DeepAuC to NCFCC and MIRCC in terms of the delay. Fig 9 shows that both DeepAuC and MIRCC experience lower delays than NCFCC because NCFCC is a window-based congestion control, where the nodes increase the number of Interest packets based on the number of received Data packets. This window-based congestion control approach is characterized by high variations in the delay. In this figure, we use solid red lines to depict the median. The data for the network delay are available with filename delay.csv [40].

## Conclusions

In this paper, we proposed a joint deep learning and auction-based caching approach in NDN to prevent congestion before it happens and avoid packet loss and high end-to-end delay. In our approach, the ISP uses collected transit traffic data to predict the future transit traffic volume. In this way, the ISP identifies overloaded transit links that may experience congestion in the future and the most requested contents that need to pass through these links. To prevent congestion, which may occur in the future on these overloaded transit links, the ISP caches identified contents to limit the transit traffic volume. However, downloading and caching paid-contents require the payment of content prices. Therefore, we proposed an auction model that helps the ISP to obtain paid-content at an optimal price, where the ISP sells cached contents to its customers. Through extensive simulation, the simulation results show that our proposal can prevent congestion, minimize end-to-end delay, and help the ISP to monetize its cache resources.
